# Rapid adaptive remote focusing microscope for sensing of volumetric neural activity

**DOI:** 10.1364/BOE.8.004369

**Published:** 2017-09-07

**Authors:** Mantas Žurauskas, Oliver Barnstedt, Maria Frade-Rodriguez, Scott Waddell, Martin J. Booth

**Affiliations:** 1 Centre for Neural Circuits and Behaviour, University of Oxford, Mansfield Road, Oxford OX1 3SR, UK; 2 Department of Engineering Science, University of Oxford, Parks Road, Oxford OX1 3PJ, UK

**Keywords:** (110.0180) Microscopy, (110.1080) Active or adaptive optics, (000.1430) Biology and medicine, (170.6900) Three-dimensional microscopy

## Abstract

The ability to record neural activity in the brain of a living organism at cellular resolution is of great importance for defining the neural circuit mechanisms that direct behavior. Here we present an adaptive two-photon microscope optimized for extraction of neural signals over volumes in intact *Drosophila* brains, even in the presence of specimen motion. High speed volume imaging was made possible through reduction of spatial resolution while maintaining the light collection efficiency of a high resolution, high numerical aperture microscope. This enabled simultaneous recording of odor-evoked calcium transients in a defined volume of mushroom body Kenyon cell bodies in a live fruit fly.

## 1. Introduction

Investigating functional connectivity between neurons in the brain is the key to understanding many fundamental questions in neuroscience. Recording neural activity with genetically encoded calcium indicators such as GCaMP [1] permits non-invasive observation of multiple neural responses, if combined with a suitable microscopic method. Yet, imaging activity quickly over large volumes inside a live brain still remains one of the greatest challenges in neuroscience microscopy [2,3]. Images from widefield single photon fluorescence microscopes can encompass a large number of neurons, but the cells of interest do not necessarily lie in one image plane. Moreover, the volumetric nature of neural tissue means that out-of-focus background often swamps useable signals. Scanning two-photon microscopy is commonly favored for imaging in scattering neural tissues as it provides high contrast, deep penetration, increased resilience to scattering and low phototoxicity to the sample. However, to compile a volume multiple planar images need to be acquired. Collecting data in this way is slow because the specimen or objective has to be moved. Imaging-induced and natural motion of the specimen are problematic because the plane of interest can be easily lost from view – our own investigations have revealed frequent translations of several micrometres when imaging neurons in the *Drosophila* brain. Single objective scanning light sheet methods could be a solution, although resolution and collection efficiency are not optimal due to the limited numerical aperture [4]. Light field microscopy can image large volumes simultaneously, but it relies on sparse labeling [5]. Two-photon fluorescence methods present the most promising avenue for fast functional imaging of densely labeled specimens. Here we have taken a new approach to fast two-photon imaging – Adaptive Optics based Volumetric Activity Sensing Two-photon (AO-VAST) microscopy – that is optimized for extraction of information from dense neural tissue.

Volumetric imaging rates can be increased by avoiding the need to move the specimen or objective. To this end, various optical axial scanning techniques have been employed. The remote-focusing principle, which is common to all of these high-speed volumetric techniques, involves shifting the imaged plane by application of a defocus phase at the pupil of the optical system. The fastest available methods rely on acousto-optical scanning for 3D random access of multiple pre-defined points within the sample [6,7]. Alternatively, spatial light modulator based multi-site excitation combined with volume projection can be used for simultaneously monitoring a subset of pre-defined cells of interest [8]. These methods are optimized for sampling of multiple spatially dispersed points, but are not practical for full volume imaging. Furthermore, the focusing is not ideal, as the quadratic phase functions introduced by the acousto-optical scanners do not perfectly match the phase required for spherical-aberration-free focusing. Systems using matched lenses and small mirror based refocusing have been used to scan in 3D at line rates of several kHz with aberration-free focusing, but the speed is still limited for complete volumes [9]. Other techniques include electrically tunable liquid lenses [10], which are slow and do not provide aberration free focusing, or ultrasound driven lenses [11], which again suffer from focusing aberrations and are limited to fast sinusoidal scanning. AO-VAST microscopy provides a combination of capabilities that cannot be achieved with these existing technologies.

## 2. Results and discussion

### 2.1 Experimental set-up

**Fig. 1:**
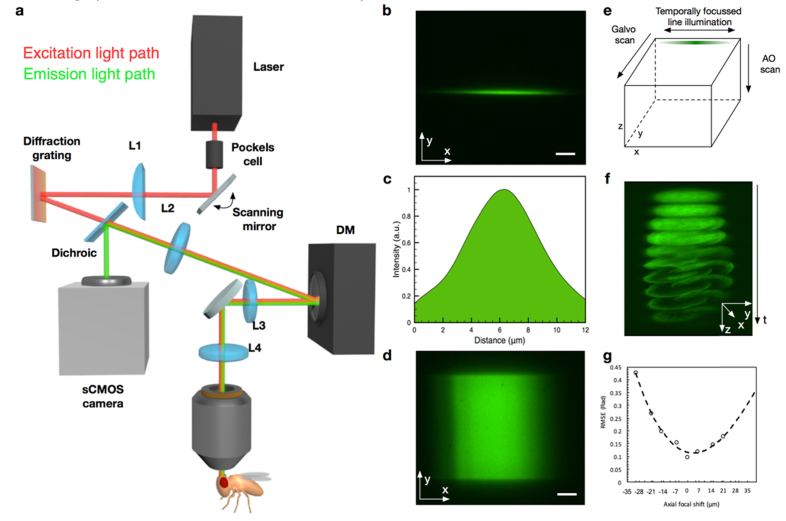
(a) Simplified schematic diagram of the AO-VAST microscope configured for in vivo imaging. (b) Illumination line exciting two-photon fluorescence in a fluorescent polymer slide indicating the shape of line illumination profile. (c) Axial profile of the excitation PSF obtained by recording fluorescence intensity when a thin layer of fluorescent dye is scanned through object plane (d) A single imaging plane obtained by laterally scanning an excitation line across a fluorescent plastic slide. (e) Volumetric imaging strategy: each optical slice is formed by scanning a line along the image plane; scanning along the optical axis is enabled by a deformable mirror. (f) Expanded 3D image of a pollen grain: the whole volume consisting of ten z-slices, each of thickness ~5 μm, was captured in 10 ms. (g) Wavefront root mean square error (RMSE) in radians for optical wavefront when remotely focusing to different axial positions. Data points are fitted with a 2nd order polynomial. In (b) and (d) the scale bar corresponds to 10 micrometers.

Measuring brain activity with high temporal resolution requires a new design philosophy to exploit different attributes of available technologies. Foremost, prioritizing the extraction of information about cell activity over the resolution of structural details leads to very different specifications for the imaging system. AO-VAST combines several technologies to enable this rapid 3D sensing of neural activity (Fig. 1(a)

). Line scanning two-photon microscopy [12] provides higher scanning speed than a point scanning approach, when employing standard off-the-shelf galvanometric mirror systems. This is combined with non-descanned detection on a sCMOS camera. Adaptive optics (AO) based remote focusing is enabled by a deformable mirror (DM), which is positioned conjugate to the objective pupil plane. The DM serves two purposes: aberration correction and shifting simultaneously the focal position of the excitation and imaging paths [13]. The line scanning strategy combined with AO axial scanning, allows speeds of up to 1000 planes, or 100 volumes per second when using strongly fluorescent samples (Fig. 1(f)).

Temporal focusing [14,15] has been used previously in the context of two-photon imaging to decouple axial and lateral confinement of excitation [16]. We took advantage of this by using temporal line focusing to match the excitation profile in the z direction to the typical size of Kenyon Cell bodies (Fig. 1(c)). Kenyon Cell somata are relatively large (up to 5 μm) compared to the resolution of a standard two-photon microscope (about 0.5 μm) and are usually distributed over a volume. Furthermore temporal focusing has been shown to provide increased robustness of the excitation beam against the effects of scattering [17]. Additionally, depending on imaging requirements, the scanning speed can be traded for a reduction of the illumination beam average power, possibly leading to reduced phototoxicity [18] to the sample.

A state of the art deformable mirror (DM) can control the wavefront at rates of up to 20 kHz [19]. This speed is more than adequate when measuring even the fastest commonly used calcium or voltage indicators [1]. The DM is also able to produce more complex shapes than the quadratic phase function that is offered by many simpler focusing methods; this permits minimization of residual wavefront errors (Fig. 1(g)). This is necessary, as refocusing in high numerical aperture systems requires a high order polynomial – not quadratic – phase function for aberration free operation. Furthermore, the DM can be used to compensate sample and system induced aberrations leading to increased signal to noise ratio (SNR) that permits shorter exposure times. DM based systems have the advantage of compactness and have a potential to exhibit lower optical loss than acousto-optical [6,7] or lens based [9] remote focusing systems with similar functionality.

In realistic practical scenarios, where labelling densities and laser power are constrained by the nature of live imaging experiments, then the speed at which the microscope can operate will be limited by the available SNR. However, SNR can be increased if the spatial resolution of the microscope is intentionally reduced so that, in effect, an image voxel measures the signal integrated over the region of interest, such as a cell body. Fast monitoring of continuous volumes can therefore be achieved by increasing the voxel size to approach the physical dimensions of a cell body, whilst maintaining the fluorescence collection efficiency of a high NA objective lens. The resulting optical sections are thicker than the sections that can be captured with standard two-photon microscopes, thus fewer images – and less time – are needed to cover the whole volume. The method also avoids the problematic loss of data due to motion of the live specimens – since movements are contained within the imaged volume, they can be dealt with through post-processing. Note that an equivalent effect cannot be achieved by using an objective lens with lower numerical aperture – while the axial resolution can be reduced this way, the fluorescence detection efficiency drops dramatically with the lower aperture angle.

**Fig. 2:**
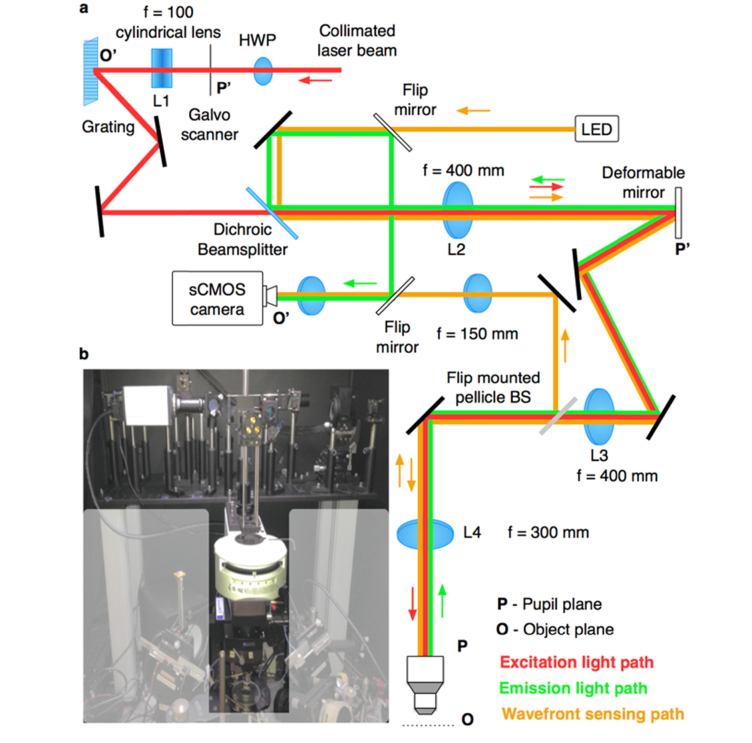
Detailed schematic of the optical set-up. O and O’ indicate object and conjugate object planes, while P and P’ indicate optical system pupil planes. The microscope can be switched to wavefront sensing mode by lowering two flip mirrors and raising a flip mounted pellicle beam splitter. (b) Photograph of the set-up. The shaded region masks instruments used for electrophysiology and odor delivery.

The optical set-up for the AO-VAST microscope (Fig. 2(a)

) was built in-house and mounted on top of a Scientifica two-photon microscope ((Fig. 2(b)). The optical power of a femtosecond excitation laser (Chameleon Ultra II, Coherent Inc.) was controlled by adjusting the voltage applied to a Pockels cell (350-80, ConOptics). The collimated laser beam was launched on a scanning mirror (GVSM001, Thorlabs) positioned at the pupil plane of the optical system, the beam was then focused with a cylindrical lens (f = 100 mm) to form a line on a grating (1200 lp/mm ruled grating, Edmund Optics), which was used to enable spatio-temporal focusing. The cylindrical lens creates an illumination beam with Gaussian intensity profile along the line (Fig. 1(d)). In future implementations of the set-up, other beam shaping optics such as Powell lens [20] or DMD based approach [21] could be used to achieve more uniform and better controlled illumination. For optimal diffraction efficiency, the laser polarization was aligned to the grating by manually adjusting the rotation of a half wave plate HWP. The light from the grating was then relayed on to a deformable mirror (DM-69, Alpao) positioned at the pupil plane of the system and re-imaged on to the objective lens (40 × , 0.8 NA water immersion LUMPLFLN 40XW, Olympus).

The fluorescence light propagating back through the same path was separated from illumination beam just after the lens L2 using a dichroic beam splitter (FF665Di02, Semrock), and de-magnified with a matched achromatic lens pair (1:3.3, Thorlabs). The image was then captured with sCMOS camera (Zyla 4.2P, Andor). When used in conjunction with 300 mm tube lens, the Olympus 40× objective produced 66.6× magnification. This image was relayed with 1:1 telescope and de-magnified 3.3 times. The resulting magnification was 20.2× . The focal lengths of optical elements in the system were chosen to produce a compact module which was compatible with the Scientifica two-photon microscope, while slightly overfilling the objective pupil with an image of the DM aperture. When necessary, camera pixel binning could be further used to reduce image sampling and gain sensitivity.

The system permits convenient switching between arc-lamp illumination (Xcite 120PCQ, Lumen Dynamics) for locating the area of interest; and two-photon illumination for fast 3D imaging by rotating the filter wheel which is built in the Scientifica two-photon microscope stand. The compact size of the custom set-up allowed it to be contained on a raised aluminum breadboard within a Faraday cage that surrounded the whole microscope.

The system resolution in *xy* was determined by a combination of several factors, in our case most critical two were: (1) dimensions of the Airy disk produced by an objective for the detection wavelength and (2) the detection pixel size. The radius of the Airy disk for the objective used in our set-up can be calculated to be 0.4 μm @ 500 nm. Using pixel binning on the camera we down-sampled the detection PSF to ~1 μm. In the *z* dimension the two-photon resolution was determined by the objective NA and temporal focusing parameters, it was measured to be ~5 μm FWHM (see Fig. 1(c)). Strictly speaking, the lateral resolution varies within the ~5 μm depth of illumination, as there is a difference between the depths of focus of the illumination and the imaging systems. The middle of the illuminated layer will be in focus at the camera, whereas those regions above and below will be out of focus. The lateral resolution will therefore vary with depth.

### 2.2 Deformable mirror training for fast remote focusing

In high numerical aperture (NA) optical systems, defocus can be described as a phase term *kz*cos(θ), where *z* is the distance between from the nominal focal plane, *k* is the wavenumber and θ is angular coordinate of a ray from the optical axis. Also, if we express the normalized radius of the pupil as ρ=sin(θ)/sin(α), where α is maximum acceptance angle of the lens, determined by its NA, then using a Taylor expansion the phase function can be rewritten as

$$\begin{array}{l} \varphi \left(\theta \right)=kz\cos\theta \\ =kz\sqrt{1-\sin^{2}\theta }\\ =kz\sqrt{1-\rho^{2}\sin^{2}\alpha }\\ \approx kz\left(1-\frac{\rho^{2}\sin^{2}\alpha }{2}-\frac{\rho^{4}\sin^{4}\alpha }{8}-\frac{\rho^{6}\sin^{6}\alpha }{16}-\ldots \right) \end{array}$$ (1)

Note that the constant term, which is a piston phase offset, can usually be ignored when considering focusing effects. For low NA optical systems, higher order terms can be neglected and the phase can be approximated as a quadratic phase $\varphi =-\frac{kz}{2}\rho^{2}\sin^{2}\alpha$. However, in high NA microscope systems, the higher order terms are essential for accurate focusing and the use of the quadratic approximation leads to the same detrimental effects as spherical aberration. AO-VAST microscopy does not rely on such approximations as the system is calibrated using the real phase induced by defocus and the deformable mirror can reproduce higher order terms.

Various fast remote focusing methods have inherent limitations preventing faithful reproduction of the defocus function. Acousto-optical scanning approximates in effect the defocus function using two acousto-optic scanners in series to introduce quadratic phase variations along *x* and *y* directions. This approach can only be extended to reproduce higher order terms through more complex arrangements that lead to increasing optical losses. Other methods such as ultrasound lenses or electrically tunable liquid lenses work by producing wave fronts that only approximate the ideal function, again leading to further residual spherical aberration.

The initial wavefront error induced by optical set-up was measured using a sensorless AO approach [22]. The wavefront error was corrected by sequentially minimizing the error induced by each orthogonal Zernike mode up to the second order spherical aberration. The best correction was achieved by using total intensity of two-photon images of thin layer of fluorescent beads. The correction obtained with sensorless AO was used as a system flat file. Similarly, by looking at the structures within the sample this approach was extended to do sample induced aberration correction. Phase maps obtained for system and sample induced aberration correction were additively combined with remote focusing phase maps to perform both functions simultaneously with a single DM.

To characterize the deviations from the ideal optical system performance [23] and to measure *in situ* the phase maps required to shift the focal plane in an aberration-free manner, we have used custom wavefront measurement based transport of intensity equation [24]. For mirror characterization the setup has to be switched to the wavefront sensing mode by lowering two flip mirrors and lifting a pellicle beamsplitter (orange path in Fig. 2) This optical arrangement provides even pupil illumination required for TIE wavefront sensing and employs a sCMOS camera for capturing the intensity distribution at the defocused pupil plane.

The wavefront sensor was used to measure the total phase introduced along the whole optical path from the LED to the sensor, which will include system aberrations, the defocus from the specimen mirror, and the phase introduced by the deformable mirror. By taking differences between phase measurements one can easily remove the system effects and determine the shape required for the DM to compensate the defocus. Note that the reflection off the specimen mirror causes cancellation of odd-symmetry aberrations and a doubling of the even-symmetry aberrations. However, this does not affect the calibration for focussing, as the defocus aberration is rotationally symmetric (even) and we take into account that a mirror displacement of *z* is equivalent to a focal displacement of 2*z*.

**Fig. 3:**
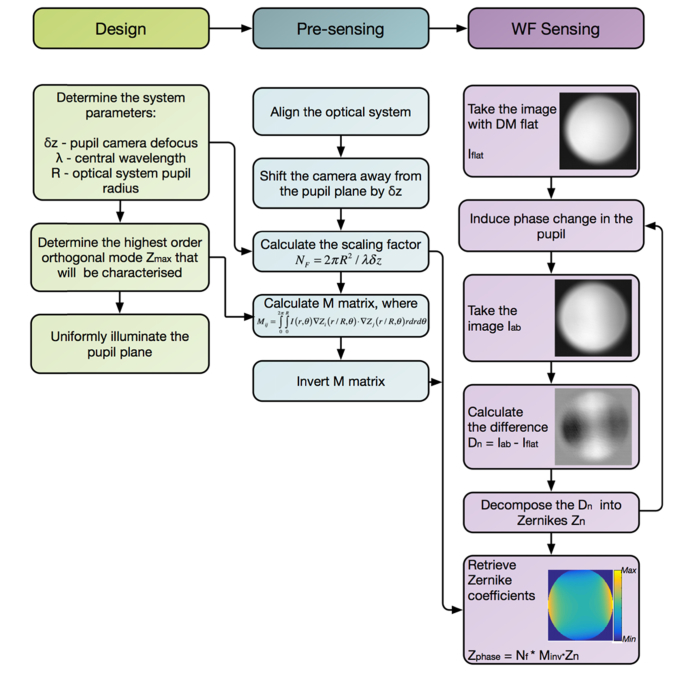
Flowchart outlining the TIE based phase retrieval for deformable mirror calibration. For the wavefront sensing, an optical pupil is evenly illuminated and the intensity maps of the light propagating through the system is captured at the plane at the slightly defocused position δ*z* away from the conjugate pupil plane. The *M* matrix and the scaling factor *N_F_* are determined by system parameters (δ*z* defocus distance, *λ* central illumination wavelength and *R* the radius of the optical pupil) and by the orthogonal mode set chosen for the wavefront decomposition (Zernike modes in this instance). Calculation of the *M* matrix is the most computationally intensive step and can be done in advance. During the wavefront sensing the difference between the images taken with a flat reference wavefront and with distorted wavefront is decomposed into chosen set of orthogonal modes *Z*_n_ and used as the input for the final equation *Z*_phase_ = N_F_*M_inv_⋅*Z*_n_.

Once a flat mirror was placed at the object plane after objective lens, a wavefront sensor as depicted in Fig. 2 was used to measure a total phase introduced by a deformable mirror and an objective lens. In the first instance the DM was trained to produce orthogonal modes by sequentially poking actuators and measuring the actuator influence functions to assemble a poke matrix. A pseudoinverse of poke matrix was then used as a control matrix, which translates desired amounts of Zernike modes into voltages that need to be applied to actuators behind the mirror membrane to impart a desired phase on the optical wavefront [25]. For flowchart outlining wavefront sensing procedure please refer to Fig. 3

.

**Fig. 4:**
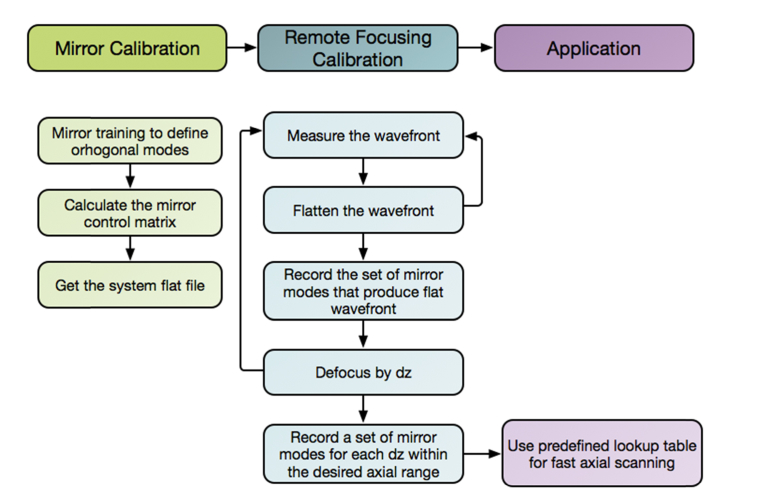
Flowchart outlining mirror training for the remote focusing algorithm.

A deformable mirror training for remote focusing was achieved by scanning objective lens focal plane relative to a flat mirror that was positioned at the object plane as outlined in Fig. 4

. Once the system was flattened for the initial position, an objective lens was slightly defocused by a small increment d*z*. A distorted wavefront was captured by the wavefront sensor and an opposite phase was applied to a DM to flatten the wavefront again. These steps were repeated for multiple positions on both sides of the focal plane to construct a lookup table of control signals that correspond to various axial focus positions.

**Fig. 5:**
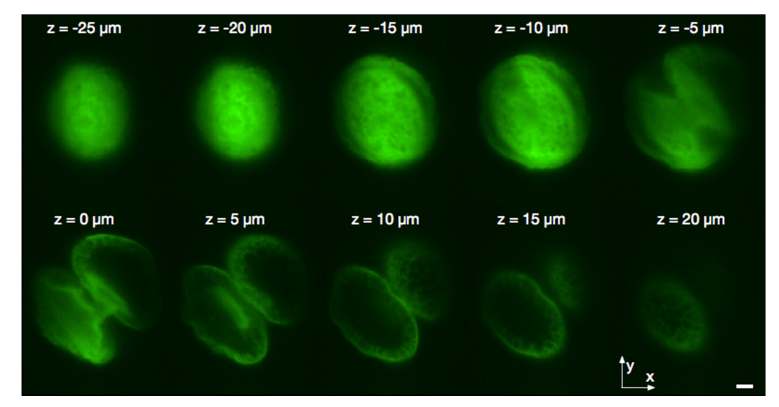
Fast imaging of a pollen grain: the whole volume consisting of ten z-slices, each of thickness ~5 μm, was captured in 10 ms. The scale bar corresponds to 10 micrometers.

A lookup table obtained during the mirror training (Fig. 4) was then used to shift the focal plane (Fig. 5

) to the required axial position at up to kHz rates. In a double pass configuration, a single DM permits refocusing for both excitation and fluorescence emission paths. These very fast imaging rates are best demonstrated when imaging brightly fluorescent samples, such as a pollen grain shown in Fig. 5, as the imaging system becomes limited by the amount of photons it can collect to form each frame.

### 2.3 In-vivo neural activity sensing

**Fig. 6:**
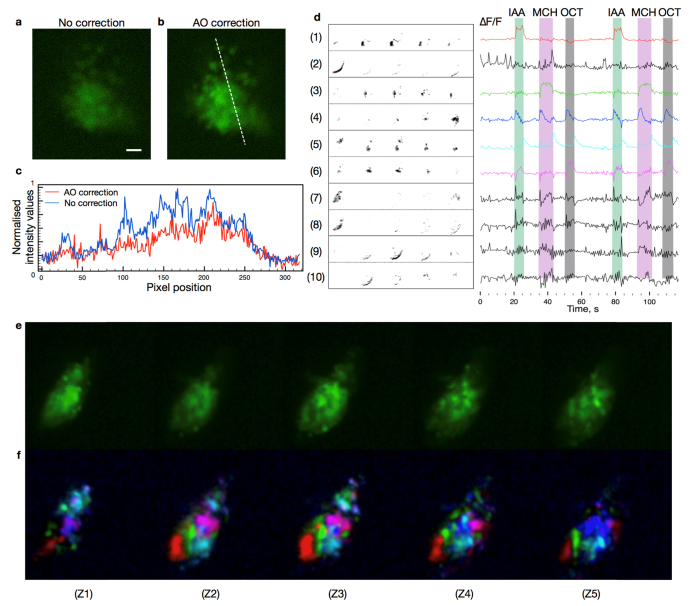
Imaging odor responses in a live fruit fly brain expressing GCaMP6f in mushroom body Kenyon Cells. (a) Image of Kenyon Cell bodies with system flat correction. Scale bar corresponds to 10 micrometers. (b) AO correction of sample induced aberrations leads to sharper images and increased image brightness. (c) Comparison of raw images acquired with and without correction of sample induced aberrations. AO correction leads to increased signal to noise ratio, permitting faster imaging. (d) Independent components extracted from a recorded video (Visualization 1) and ∆F/F responses corresponding to each independent component. Green, purple and grey color bars represent the time periods where the fly was exposed to IAA, MCH and OCT odors. (e) Averaged recording of Kenyon Cells expressing GCaMP6f representing cumulative fluorescence throughout full odor delivery sequence. Each image going from left to right corresponds to a 50 × 80 μm consecutive *z*-slice separated by 5 μm. (f) A composite image where different colors correspond to spatial filters 1,3,4,5,6 in (d) 3D rendering is also available in Visualization 3.

Combined, these technologies can deliver fast and robust neural activity sensing *in vivo*. We imaged activity in somata of the Kenyon cells in the mushroom body in awake *Drosophila* using the genetically encoded calcium indicator GCaMP6f (Fig. 6

). Rapid monitoring of Kenyon cell body activity is difficult with current single plane imaging techniques, as the cell bodies are distributed throughout the volume and they respond simultaneously in different axial positions.

For live imaging we used LexAop-GCaMP6f; 247-LexA::VP16 flies [26,27]. Three- to eight-day-old flies were briefly anesthetized on ice and mounted in a custom chamber. The head capsule was opened under room temperature carbogenated (95% O_2_, 5% CO_2_) buffer solution (103 mM NaCl, 3 mM KCl, 5 mM N-Tris, 10 mM trehalose, 10 mM glucose, 7 mM sucrose, 26 mM NaHCO3,1 mM NaH_2_PO_4_, 1.5 mM CaCl_2_, 4 mM MgCl_2_, osmolarity 275 mOsm [pH 7.3]). The legs and proboscis were immobilized with wax. For the odor response imaging, three different odors were delivered on a clean air carrier stream using a custom-designed system [26]. 5 seconds of IAA (isoamyl acetate) were followed by 12 seconds of air, then 8 seconds of MCH (methylcyclohexanol), then 10 seconds of air, and finally 5 seconds of OCT (octanol).

As a demonstration of capability, we imaged a volume of 50 × 80 × 20 μm consisting of a set of 5 axial slices (each about 5 μm thick) separated by 5 μm. The imaging rate was limited by the sample brightness to 2 volumes per second. The instrument design meant that no gaps existed between adjacent sections. The length of the focal line was chosen as a compromise between field of view and the laser power that was necessary to obtain suitable fluorescence emission. The line length could be increased if higher laser power is available. Alternatively, a larger area could be accessed with the same laser power by using a scanned point array.

Excitation power was set to 100 mW average power spread across the excitation line (Fig. 1(c)). During imaging, the objective and the sample were kept in fixed positions. Even though there were obvious movements during acquisition, no pre-processing for movement correction was performed to demonstrate the robustness of the method. The approach could be extended to more extreme movements by pre-processing to realign the images in three dimensions.

We also extended automated cellular signals analysis methods [28,29] to work with volumetric data to identify the groups of cells that respond together during the stimulation. The resulting ∆F/F traces (Fig. 6) show that our fast sensing method can reproducibly capture the responses of cell groups that respond to different odor stimuli. Furthermore, initial results indicate that our method may be used for long periods without causing significant phototoxicity to the fly. A video (Visualization 2) shows a fly responding to odor stimuli while Kenyon Cell bodies were imaged in different locations with laser power varying from 70 mW to 140 mW for over 25 minutes. We have noticed that, depending on the quality of sample preparation and the power of laser excitation, the imaging time can be easily extended up to an hour. Defining the limits will require more experiments.

## 3. Conclusion

The AO-VAST sensing approach enables monitoring of neural activity throughout continuous volumes. The combination of temporal focus line scanning two-photon excitation and AO based remote focusing permits adjustable reduction of spatial sampling over the volume in order to increase temporal resolution. As a result of these resolution compromises, one can extract enough information to determine volumetric activity patterns. While the speed of live demonstrations was limited by the brightness of the specimens used (volume rates of 2 Hz), we have demonstrated that the optical system itself is capable of volumetric imaging at rates of up to 100 Hz. These rates are not achievable for full volumetric imaging with standard point scanning two-photon imaging modalities.

System performance in SNR-limited samples can be further optimized by refining the illumination pattern to obtain more efficient time-averaged two photon emission [30]. Widefield detection of fluorescence can lead to increased background signal when imaging deeper through scattering tissue or in even more scattering samples. This can be improved by using a synchronized digital confocal slit using the rolling shutter mode of the sCMOS camera. Confocalisation of the system would also lead to improved resolution along line scanning direction. Furthermore, the DM used for fast remote focusing, can be simultaneously employed to cancel sample induced aberrations and further increase SNR to boost practical imaging rates. Lastly, the compact footprint of the system allows it to be easily added to commercial two-photon microscopes.

## Disclosures

The authors declare that there are no conflicts of interest related to this article.
